# Combining Neural Architecture Search and Weight Reshaping for Optimized Embedded Classifiers in Multisensory Glove

**DOI:** 10.3390/s25196142

**Published:** 2025-10-04

**Authors:** Hiba Al Youssef, Sara Awada, Mohamad Raad, Maurizio Valle, Ali Ibrahim

**Affiliations:** 1 Department of Computer and Communication Engineering, Lebanese International University, Beirut 1105, Lebanon; hiba.youssef@liu.edu.lb (H.A.Y.);; 2 Department of Naval, Electrical, Electronics and Telecommunications Engineering, University of Genoa, 16126 Genoa, Italy

**Keywords:** Neural Architecture Search, weight reshaping, quantization, multisensory glove

## Abstract

Intelligent sensing systems are increasingly used in wearable devices, enabling advanced tasks across various application domains including robotics and human–machine interaction. Ensuring these systems are energy autonomous is highly demanded, despite strict constraints on power, memory and processing resources. To meet these requirements, embedded neural networks must be optimized to achieve a balance between accuracy and efficiency. This paper presents an integrated approach that combines Hardware-Aware Neural Architecture Search (HW-NAS) with optimization techniques—weight reshaping, quantization, and their combination—to develop efficient classifiers for a multisensory glove. HW-NAS automatically derives 1D-CNN models tailored to the NUCLEO-F401RE board, while the additional optimization further reduces model size, memory usage, and latency. Across three datasets, the optimized models not only improve classification accuracy but also deliver an average reduction of 75% in inference time, 69% in flash memory, and more than 45% in RAM compared to NAS-only baselines. These results highlight the effectiveness of integrating NAS with optimization techniques, paving the way towards energy-autonomous wearable systems.

## 1. Introduction

The rapid development of wearable technologies has brought increasing attention to energy autonomous systems capable of operating continuously without reliance on frequent recharging or external power sources [1]. Among these, multisensory gloves represent a promising class of devices with applications in healthcare monitoring, rehabilitation, and human–computer interaction. Self-powered smart gloves utilizing triboelectric nanogenerators (TENGs) have been developed for real-time gesture recognition, demonstrating the feasibility of energy-autonomous wearable devices [2]. Separately, rehabilitation-oriented multisensory gloves, such as those in the MIDAS system, integrate tactile, visual, auditory, and olfactory feedback to enhance patient motivation and engagement during hand therapy [3]. Ensuring energy autonomy in such systems is crucial to improve usability, reduce maintenance costs, and enable long-term deployment in pervasive computing scenarios [4,5].

Achieving energy autonomy in wearable gloves typically requires deploying neural networks on resource-constrained microcontrollers (MCUs), enabling the gloves to intelligently recognize gestures and adapt to user interactions in real time. While these platforms provide ultra-low power consumption, they are inherently limited in processing power, memory, and storage [6]. This limitation presents a major challenge for implementing embedded neural networks (NNs) for applications such as gesture recognition or texture classification, as conventional architectures are often too complex to run efficiently on MCUs. Balancing performance, accuracy, and resource usage therefore makes the development of optimized neural architectures a critical task [7].

In recent years, Neural Architecture Search (NAS) has emerged as a powerful approach for automating the design of neural networks under strict resource constraints. By exploring a search space of candidate models, NAS can identify architectures that balance accuracy with computational efficiency, thus facilitating deployment on microcontrollers and other edge devices [8,9]. However, while NAS provides a systematic framework for architecture optimization, the computational complexity and resource footprint of the resulting models can still hinder their practical deployment in energy autonomous systems. To address this, recent research has extended NAS beyond architecture design alone by embedding compression techniques such as pruning and quantization directly into the search process. In these joint optimization approaches, the search algorithm treats both the network structure (number of layers, filter sizes, connectivity) and the compression policy (sparsity level, quantization bitwidth) as simultaneous decision variables. For example, APQ performs a unified search over architectures, pruning schemes, and quantization strategies, showing improvements over pipelines that apply NAS, pruning, and quantization separately [10]. Similarly, HQNAS (Hardware-Aware Quantized Neural Architecture Search) incorporates quantization bitwidth into the NAS search space to produce efficient hardware-aware designs [11]. Other methods, such as DJPQ (Differentiable Joint Pruning and Quantization), jointly optimize pruning masks and quantization during search, achieving large reductions in bit-operations while maintaining accuracy [12]. While these joint strategies can yield highly compact and accurate models, they significantly enlarge the search space, making training and exploration computationally expensive. In addition, the resulting models may be less flexible to adapt across different hardware platforms or application scenarios.

In contrast, the strategy adopted in this work follows a modular, step-by-step pipeline that integrates NAS with optimization techniques tailored for energy autonomous multisensory gloves. It reduces search complexity and enables more efficient exploration of architectures. Moreover, it offers greater flexibility, since compression levels, quantization precision, or even optimization strategies can be adjusted post-search without the need to re-run the entire NAS process. This modularity also enhances portability across hardware platforms, making the approach practical for diverse low-power deployment scenarios. The main contributions of this paper can be summarized as follows:

1. Applying Hardware-Aware NAS (HW-NAS) to automatically derive an optimized 1D-CNN architecture that satisfies the hardware constraints of the target low-power device while simultaneously achieving high accuracy (average up to 96.67% overall datasets).
2. Employing additional optimization strategies, including weight reshaping, quantization, and their combination, to further reduce the model complexity while improving performance. These refinements achieve an average reduction of 69% in flash memory and 45% in SRAM compared to the baseline NAS-generated models.
3. Validating robustness and generalization by evaluating the proposed approach on three distinct datasets. Experimental results demonstrate consistent improvements, with average classification accuracy up to 96.78% across tasks, thereby confirming the effectiveness of the proposed method in diverse scenarios.

## 2. Related Works

In recent years, significant efforts have been made to develop neural networks that achieve high performance while remaining computationally efficient and suitable for deployment on resource-constrained devices [8]. Among these efforts, manually designed lightweight models such as MobileNets [8], SqueezeNet [13], and ShuffleNets [14] have emerged. Nevertheless, manually identifying the optimal neural network architecture remains a time-consuming process that demands substantial human effort [15]. Consequently, research attention has shifted toward methods that automate the design process. Neural Architecture Search (NAS) is a subfield of machine learning and artificial intelligence that addresses this challenge by automating the generation and optimization of neural network architectures [16]. Various search strategies have been proposed in NAS, including reinforcement learning (RL), evolutionary algorithms (EAs), Bayesian optimization (BO), and gradient-based approaches, to identify the optimal architecture while adhering to the constraints of the target hardware. In the RL approach, the controller iteratively proposes architectures, receives validation performance as a reward signal, and uses that feedback to improve future proposals. This method reduces reliance on manual design and automates the discovery of high-performing networks [17]. Notable examples of this strategy include FPNet [18], Codesign-NAS [19], and MNASNet [20]. EA-based methods evolve populations of architectures over successive generations using selection, mutation, and crossover operators, promoting architectural diversity [21]. BO-based NAS employs probabilistic models to predict performance and balance exploration and exploitation, enabling efficient search [22]. Gradient-based NAS allows continuous optimization of architecture parameters using gradient descent, significantly reducing computational costs [23,24]. Overall, NAS represents a significant advancement in automated machine learning, providing a systematic and efficient methodology to discover high-performing neural architectures that often surpass manually designed networks. While NAS effectively discovers high-performing architectures, deploying these networks on resource-constrained devices often requires additional optimization to reduce computational cost and energy consumption.

Several well-established optimization techniques have been proposed to address this challenge. Among these, model compression and pruning are among the most widely used strategies. Pruning eliminates redundant weights, neurons, and even entire channels, leading to more compact and faster models while maintaining comparable accuracy [25]. Depending on the strategy, pruning can be unstructured [26], which eliminates individual weights, or structured [27], which discards filters, layers, or blocks in a way that better aligns with hardware efficiency. Quantization reduces the numerical precision of weights and activations, commonly from 32-bit floating point to 8-bit integer [28] or even binary formats [29], which lowers memory requirements and accelerates inference. More advanced methods employ mixed-precision quantization, where different layers are assigned different bit-widths depending on their sensitivity to accuracy loss [30]. CLADO (Cross Layer Dependency-Aware Optimization) models cross-layer quantization error and reduces MPQ (Mixed Precision Quantization) to a small IQP (Integer Quadratic Program), enabling fast, cross layer-aware bit-width assignment that outperforms layer independent sensitivity heuristics [31]. FLIQS (Floating-Point and Integer Quantization Search) proposes the first one-shot mixed precision search that finds integer and low-precision floating-point allocations without retraining and also shows that joint quantization with architecture search can improve ImageNet accuracy on MobileNetV2 search spaces [32]. QBitOpt (optimal bit-width allocations during QAT) formulates bit-width reallocation as a constrained optimization solved during QAT (Quantization-Aware Training), giving provable satisfaction of resource constraints and strong ImageNet results for mixed-precision networks [33]. Finally, hardware-aware optimization [34] explicitly incorporates device-specific constraints such as latency, memory bandwidth, and energy usage into the design or training process, ensuring that models are not only accurate but also practical for real-world deployment on mobile and embedded systems. An emerging research direction incorporates pruning and quantization directly into the NAS process, enabling a unified optimization framework in which architectural design and efficiency considerations are addressed simultaneously. For instance, APQ jointly searches for architecture, pruning, and quantization policies, optimizing all components simultaneously [10]. QuantNAS incorporates quantization-awareness directly into NAS, producing architectures suitable for efficient mobile deployment [35]. Pruning-as-Search (PaS) integrates channel pruning within NAS, yielding compact, high-performing networks [36]. Together, these methods illustrate a unified framework in which architecture design and optimization strategies are co-explored, effectively balancing accuracy, efficiency, and deployability.

Current approaches such as APQ [10], QuantNAS [35], and PaS [36] jointly optimize architecture, pruning, and quantization within the NAS process, achieving efficiency gains but creating a very large search space, leading to high computational cost and long evaluation time for candidate models. Additionally, this approach reduces flexibility, as adapting the resulting model to new hardware often requires repeating the full search. In contrast, a decoupled strategy was proposed: performing hardware-aware NAS first, followed by targeted post-hoc optimization. This approach reduces search complexity, preserves the flexibility to fine-tune models for specific hardware constraints, and produces efficient, high-accuracy networks suitable for deployment on resource-constrained devices.

## 3. Methodology

**Figure 1:**
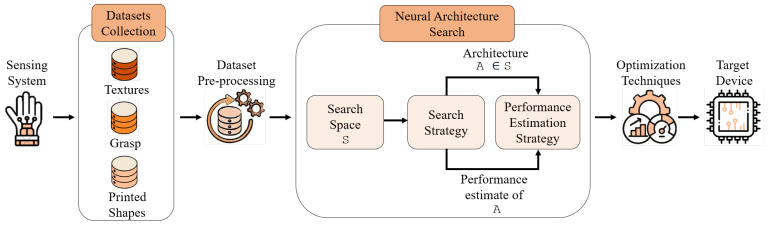
Proposed framework architecture.

The proposed workflow, illustrated in Figure 1, begins with the sensing system used in this work, which is employed to collect three distinct datasets. The datasets are then prepared through cleaning, normalization, and other pre-processing operations to ensure data quality and consistency. A 1D-CNN model is selected for this application because it effectively captures temporal and local patterns in sequential signals, while remaining computationally efficient, an essential factor for embedded deployments [37]. Once the data are prepared, HW-NAS is applied to automatically explore and evaluate the candidate 1D-CNN model architectures within a predefined search space, guided by performance metrics and constraints of the target hardware. The target deployment device is the NUCLEO-F401RE board, sourced from STMicroelectronics, which provides 512 KB of flash memory and 96 KB of SRAM, necessitating careful consideration of model size and memory usage. The selected architectures are then refined using optimization techniques to enhance performance while meeting resource limitations. Finally, the optimized model is deployed on the target board, ensuring that the solution meets the computational requirements.

### 3.1. Sensing System

**Figure 2:**
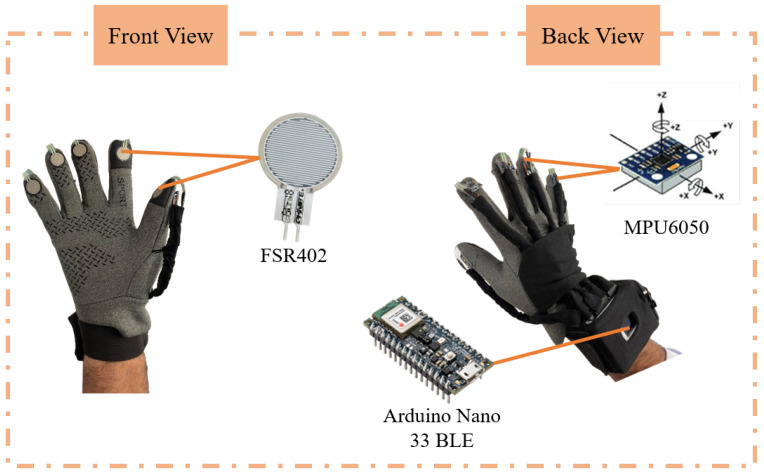
Multisensory glove.

Sensing systems play a crucial role in allowing robots and wearable devices to detect and respond to their surroundings, supporting activities like object manipulation, navigation, and human–machine interaction. Advances in tactile sensing have further improved these systems, enabling more natural, precise, and responsive interactions [38]. The multisensory glove introduced in [39] is used in this study. As shown in Figure 2, the setup consists of a commercial glove integrated with five Force Sensing Resistor (FSR402) sensors and five Inertial Measurement Units (MPU6050). Each finger is equipped with an IMU mounted on the back of the distal phalanx, containing a three-axis accelerometer and a three-axis gyroscope. Correspondingly, an FSR402 sensor is placed on the front of each finger. All sensors interface with an Arduino Nano 33 BLE via a custom PCB using the I2C protocol, with data sampled at 200 Hz. A graphical user interface (GUI) developed in LabVIEW that supports data acquisition, visualization, and system testing was used to collect the datasets.

### 3.2. Datasets

**Figure 3:**
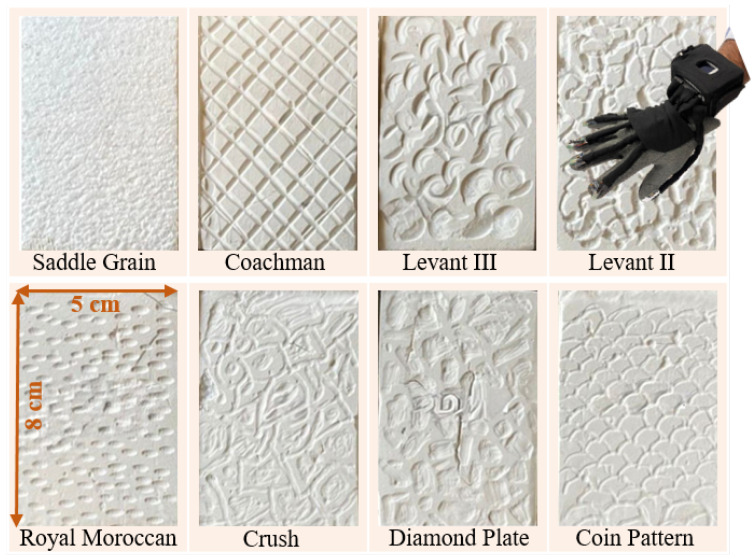
*Textures* dataset collection.

In this work, three distinct datasets, *Textures*, *Grasp* and *Printed Shapes*, were utilized to evaluate the proposed approach and ensure comprehensive validation across varying data characteristics. These datasets, collected using the same sensing glove, vary in terms of size, complexity, and data distribution, providing a comprehensive benchmark for assessing accuracy, robustness, and generalization. The *Textures* dataset involved eight distinct textures, “Saddle Grain”, “Coachman”, “Levant III”, “Levant II”, “Royal Moroccan”, “Crush”, “Diamond Plate”, and “Coin Pattern”, affixed to a table. The participants were instructed to wear the sensing glove, slide their hand over the texture, and subsequently lift it as shown in Figure 3. This interaction sequence was performed 70 times for each texture, resulting in a total of 560 recorded trials. This dataset can be defined as $D_{1}=\left\{{\left(X,y\right)}_{i},\;X_{i}\in R^{N_{C}\times N_{S}},\;y_{i}\in \left\{SaddleGrain,\ldots ,CoinPattern\right\};\;i=1,\ldots ,560\right\}$.

**Figure 4:**
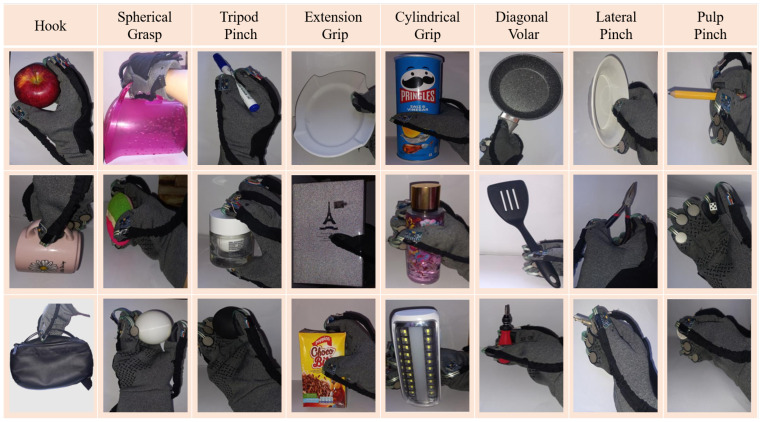
Grasp dataset collection.

The *Grasp* dataset included 24 distinct objects (8 categories with 3 objects each, as listed in Figure 4). The participants randomly grasped these objects 70 times per object, resulting in 1680 trials. This dataset is defined as $D_{2}{=\{\left(X,y\right)}_{i},\;X_{i}\in R^{N_{C}\times N_{S}},$$\;y_{i}\in \left\{PerfumeBottle,\ldots ,Marker\right\};\;i=1,\ldots ,1680\}$.

**Figure 5:**
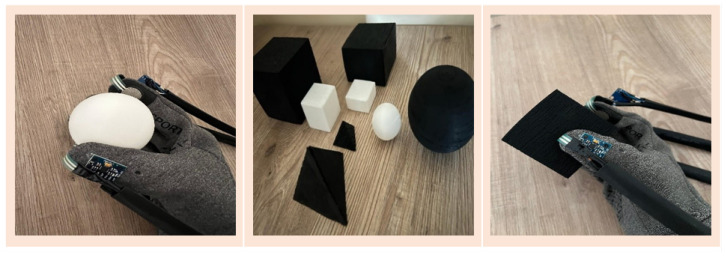
*Printed Shapes* dataset collection.

For the *Printed Shapes* dataset of [39], 16 objects were 3D-printed in four distinct shapes, cube (C), parallelepiped (P), tetrahedron (T), and sphere (S), with each shape produced in two sizes: large (L) and small (S). In addition, each shape included one hard (H) and one soft (S) version. This combination of shape, size, and stiffness resulted in the following object set = {PLH, PLS, PSH, PSS, CLH, CLS, CSH, CSS, TLH, TLS, TSH, TSS, SLH, SLS, SSH, SSS}. For data collection, five participants wore the sensing glove and interacted with the set of objects through a sequence of grasping, holding, and releasing actions performed naturally within 1 second, as illustrated in Figure 5. Each subject repeated the sequence 30 times per object, yielding 2400 trials. This dataset can be formalized as follows: $D_{3}=\left\{{\left(X,y\right)}_{i},\;X_{i}\in R^{N_{C}\times N_{S}},\;y_{i}\in \left\{PLH,PLS,\ldots ,SSS\right\};\;i=1,\ldots ,2400\right\}$.

Where $N_{c}$ represents the number of channels and $N_{s}$ denotes the number of samples per channel across the three datasets.

The pre-processing workflow is applied uniformly across the three datasets, each structured with samples grouped into folders representing distinct classes. For every sample, a fixed-size segment is extracted to ensure consistent input dimensions, adjusted according to the specific shape of each dataset. To maintain a balanced dataset and avoid class bias, a maximum limit is imposed on the number of samples per class. All input features are normalized to the [0, 1] range using min-max scaling, which helps stabilize and speed up the training process. To address the potential numerical instability caused by features with constant values, appropriate measures are implemented during normalization to prevent division by zero errors. The class labels are transformed into single encoded vectors for multi-class classification purposes. Finally, the datasets were divided into three subsets each, 70% for training, 15% for validation, and 15% for testing, with proportional sampling to preserve the class distribution across all splits. The *Textures* dataset contains 392 training, 84 validation, and 84 test samples; the *Grasp* dataset contains 1680 training, 360 validation, and 360 test samples; and the *Printed Shapes* dataset contains 1176 training, 252 validation, and 252 test samples. No additional filtering was applied during pre-processing to maintain consistency with the raw sensor data expected in real-time applications.

### 3.3. Neural Architecture Search

The rise of Neural Architecture Search (NAS) has been driven by the increasing complexity and demands of deep neural networks (DNNs). The performance of a deep learning model on a given task is strongly influenced by the structure and complexity of its architecture. Designing an optimal architecture manually can be difficult and time-consuming, prompting the need for automated solutions. NAS aims to tackle this problem by leveraging machine learning to automate the architecture design process. NAS approaches are generally categorized along three main dimensions, as illustrated in Figure 1: the search space, the search strategy, and the performance estimation strategy. The search strategy employs random sampling to select an architecture A from the predefined search space S. While more sophisticated methods exist, random sampling provides a computationally efficient and unbiased approach for exploring the search space. This selected architecture is then evaluated by the performance estimation strategy, which provides an estimated performance score of A back to the search strategy.

### 3.4. Data Pre-Processing

#### 3.4.1. Search Space

HW-NAS aims to search the high-performing neural network architectures tailored for deployment on specific target hardware by exploring a predefined search space S. In this work, validation accuracy is adopted as the primary metric to evaluate the 1D-CNN candidate architecture. The search is conducted within a constrained hyperparameter space (see Table 1), selected based on the literature [40,41]; it includes the number of convolutional layers, filter sizes, kernel dimensions, pooling strategies, and similar regularization and training parameters. These constraints promote the exploration of diverse models while balancing performance and complexity.

#### 3.4.2. Search and Performance Estimation Strategies

**Figure 6:**
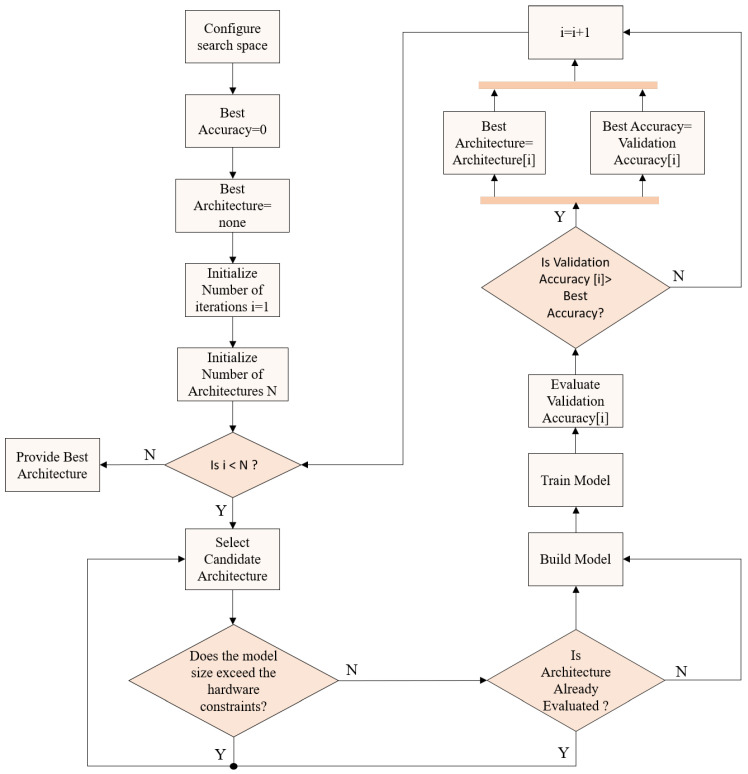
NAS search strategy flow.

The proposed search strategy, as outlined in the flowchart of Figure 6, begins by configuring the search space, which defines all possible neural network architectures to be explored. Initially, the iteration counter is initialized alongside the total number of architectures to be evaluated. In each iteration, a candidate architecture is selected from the search space, and a preliminary check is performed to ensure that its size does not exceed the hardware limitations of the target device. If the architecture meets these constraints, a second check verifies whether it has already been evaluated previously, thereby avoiding redundant computations. If the architecture is new, it is built and trained on the training dataset before being evaluated on the validation set to obtain its validation accuracy. This accuracy is then compared with the current best accuracy, and if it is higher, both the best accuracy and the best architecture are updated accordingly. The iteration counter is then incremented, and the process is repeated until the specified number of architectures has been evaluated. Finally, the five architectures that achieve the highest validation accuracies are returned as the top 5 models. This method ensures systematic exploration of the search space while eliminating duplicate evaluations and selects the best architectures based on validation performance.

#### 3.4.3. Early Stopping Strategy

In resource-constrained Neural Architecture Search (NAS), especially when targeting tiny networks with few parameters, full training of each candidate model becomes inefficient as the majority of performance trends can be inferred within the early stages of training. At the same time, one of the main challenges in deep learning is overfitting, which occurs when a model performs well on the training data but poorly on new, unseen data. Limited dataset size and diversity can lead to poor generalization, causing high training accuracy but low accuracy on test and real-world data. Increasing the number of training epochs can also contribute to overfitting, as the model continues to learn from training data, typically increasing training accuracy but potentially harming generalization [42,43].

Unlike classical training strategies that use a fixed number of epochs for all model configurations, our method incorporates an early stopping strategy to dynamically determine the optimal number of training epochs for each sampled architecture. This strategy is integrated into the feedforward and backpropagation loops during the model training process. Rather than relying on manually selected epoch values, the early stopping monitors the validation accuracy of each model and stops training once no further improvement is observed, thereby avoiding overfitting and unnecessary computation. To ensure that early stopping does not stop prematurely due to random fluctuations in validation performance, a patience threshold is defined. This threshold allows the model to continue training for a number of epochs even after reaching a plateau, giving it the opportunity to recover and improve if possible. Additionally, a learning rate scheduler is applied in parallel to progressively reduce the learning rate when the performance stabilizes, allowing finer convergence before training is terminated. Together, these components eliminate the need for fixed training durations and allow the model to autonomously determine how long to train based on performance signals.

This approach includes two levels of control. On the one hand, the macro-level controller governs when to stop training an entire model configuration, using the early stopping callback based on the validation accuracy. On the other hand, the micro-level controller operates during training by adapting the learning rate within each architecture training phase to encourage further learning before deciding to stop. This combined control ensures that the model stops training only when further progress is unlikely, improving both training efficiency and model generalization.

However, such early termination may limit the model exposure to potentially useful data patterns that would be seen in later epochs. To mitigate this limitation, the best architecture identified during the search is retrained on the union of training and validation data. This final training phase allows the model to make use of all available labeled data while still benefiting from the optimized training duration determined earlier. This strategy balances training efficiency with model robustness and leads to improved test-time generalization.

### 3.5. Optimization Techniques

The optimal 1D-CNN models identified through NAS for each dataset served as the starting point for further efficiency improvements. Specifically, the focus was on developing models that are smaller, faster, and more practical for real-world deployment while maintaining high accuracy. To achieve this, three optimization techniques have been applied. The first technique involved weight reshaping, where unnecessary weights were removed and the remaining ones were reorganized. Subsequently, the model structure was adjusted to improve efficiency. The second technique, integer quantization, reduces the precision of model parameters to save memory and accelerate inference. Finally, the two methods were combined, aiming to maximize overall benefits. Each technique was applied individually and in combination across the three dataset-specific models, allowing us to extend optimization beyond the initial architecture search. The following sections provide an overview of these methods.

#### 3.5.1. Weight Reshaping Method

In this study, a post-pruning weight restructuring approach was applied to optimize three 1D-CNN architectures. This method converted unstructured sparsity, introduced by magnitude-based pruning using the TensorFlow Model Optimization Toolkit (TFMOT) [44], into a structured model format suitable for efficient deployment on resource-constrained hardware.

The process began with unstructured weight pruning, where 50% of the weights in each layer were removed during training using a constant sparsity schedule. This level of sparsity was chosen as a balance between reducing model size and maintaining task performance [45]. After pruning, the model was stripped of its pruning wrappers, leaving a sparse but structurally unchanged network. Zero-valued weights were then removed from the matrices of each layer, and the remaining non-zero elements were compressed into flattened vectors. These vectors were reshaped to fit smaller layer dimensions, and the model was rebuilt to match these compact weight sets.

Building on this, the core of the method involved layer-wise restructuring of the pruned model into a more compact architecture. For convolutional layers, the number of filters was typically halved based on their position within the model to reflect the reduced parameter count, with subsequent layers adjusted accordingly to ensure compatibility in input and output dimensions. Dense layers underwent a similar reduction in intermediate units, with their shapes adapted to match the output of preceding layers; however, the final output layer was preserved to maintain the original output dimensions and task performance. Other layers, such as Flatten, Dropout, Batch Normalization, and Pooling layers, were retained without modification, since they do not contain trainable parameters and thus do not impact model size.

Following the layer-wise restructuring, the optimized model was fully reconstructed and underwent fine-tuning with appropriate hyperparameters, preserving its original functionality, improving deployment efficiency, and recovering any potential loss in accuracy.

In relation to conventional pruning approaches, unstructured pruning preserves accuracy but produces irregular weight patterns that limit hardware efficiency, whereas structured (organized) pruning removes entire filters or channels but can lead to larger accuracy loss under high compression. The proposed method leverages the advantage of both approaches. First, unstructured pruning is applied to identify a sparse, high-accuracy network. Then, the pruned weights are reorganized through layer-wise restructuring into a compact, structured configuration. This combination enables the network to maintain task performance while producing a hardware-efficient architecture.

#### 3.5.2. Quantization

In parallel, a post-training integer quantization was applied to further compress the 1D-CNN models and improve inference efficiency. This technique converted pre-trained floating-point weights and activations into 8-bit integer representations, a widely used precision that balances memory savings and computational speed with minimal impact on accuracy [46]. Quantization was performed using a TensorFlow Lite post-training framework [47], which calibrated the model on a representative dataset to ensure that the reduced precision minimally affected model outputs. While slight reductions in accuracy may occur, this method provides a practical trade-off between model size and performance, making it particularly suitable for deployment on resource-constrained hardware.

#### 3.5.3. Combined Weight Reshaping and Quantization

To further enhance model efficiency, the weight-reshaped models were subsequently subjected to post-training integer quantization. This sequential application leveraged the structural simplification achieved through pruning and reshaping, followed by precision reduction, resulting in models that are compact and computationally efficient. Together, these techniques enabled the deployment of high-performance 1D-CNN models on resource-constrained hardware platforms.

To sum up, the optimization pipeline comprised three stages: weight reshaping through pruning and layer restructuring, post-training integer quantization, and a combined approach applying both sequentially. These steps were integrated to yield compact and efficient 1D-CNN models, which were evaluated in the subsequent section.

## 4. Results and Discussion

### 4.1. Evaluation of NAS

This section reports the best 1D-CNN architectures obtained through NAS along with their classification results for the three datasets: *Textures*, *Grasp*, and *Printed Shapes*. For each dataset, the NAS process evaluated multiple candidate architectures and selected the top five models with the highest validation performance while taking hardware constraints into account. Table 2 presents the architecture of the best model among these five for each dataset. For the *Textures* dataset, the best model employs two convolution layers with 128 filters and a kernel size of 2, without any dense layers. The network uses ReLU activation and a very small L2 regularization. In contrast, for both the *Grasp* and *Printed Shapes* datasets, the NAS process converged to the same architecture, consisting of two convolution layers with 64 filters each, a kernel size of 4, average pooling, and Tanh activation, also without dense layers. This outcome indicates that these tasks can be effectively addressed through convolution feature extraction alone. The observed differences in filter size, pooling strategy, and activation function highlight how NAS tailors the architecture to the specific demands of each dataset.

**Figure 7:**
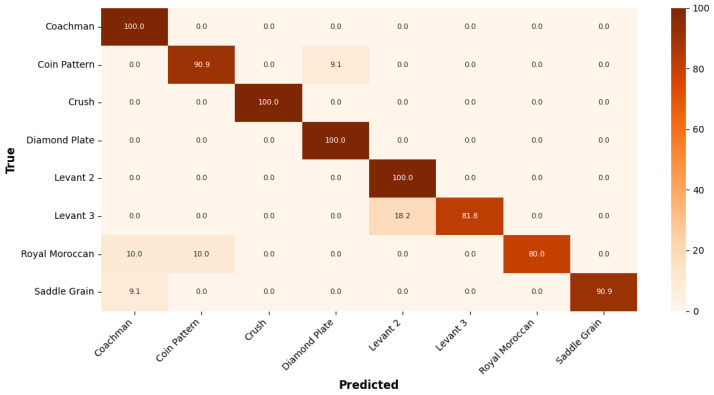
Confusion matrix of the *Textures* dataset classification performance.

The classification performance is illustrated using confusion matrices. For the *Textures* dataset, as shown in Figure 7, the NAS selected architecture achieved its highest classification accuracy of 100% for several classes, indicating a strong ability to distinguish those texture patterns. The lowest accuracy was 80%, observed for the *Royal Moroccan* class. Overall, the model demonstrates high recognition capability with only minor confusion between certain texture types.

**Figure 8:**
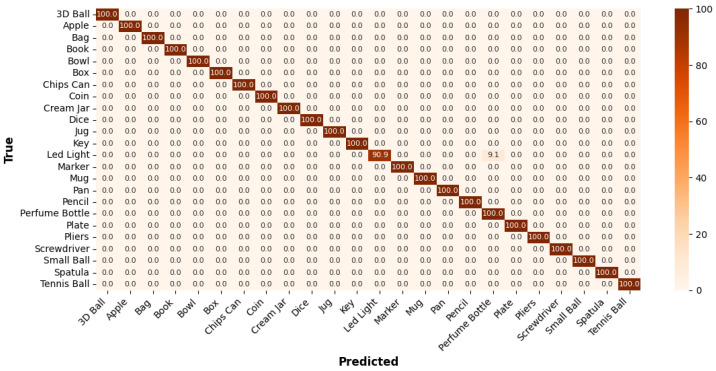
Confusion matrix of the *Grasp* dataset classification performance.

Figure 8 presents the confusion matrix for the *Grasp* dataset, illustrating that the model achieves high accuracy of 100%. However, the *Led Light* shows the lowest accuracy at 90.0%, often being misclassified as *Perfume Bottle* highlighting challenges in distinguishing objects with similar shapes and sizes.

**Figure 9:**
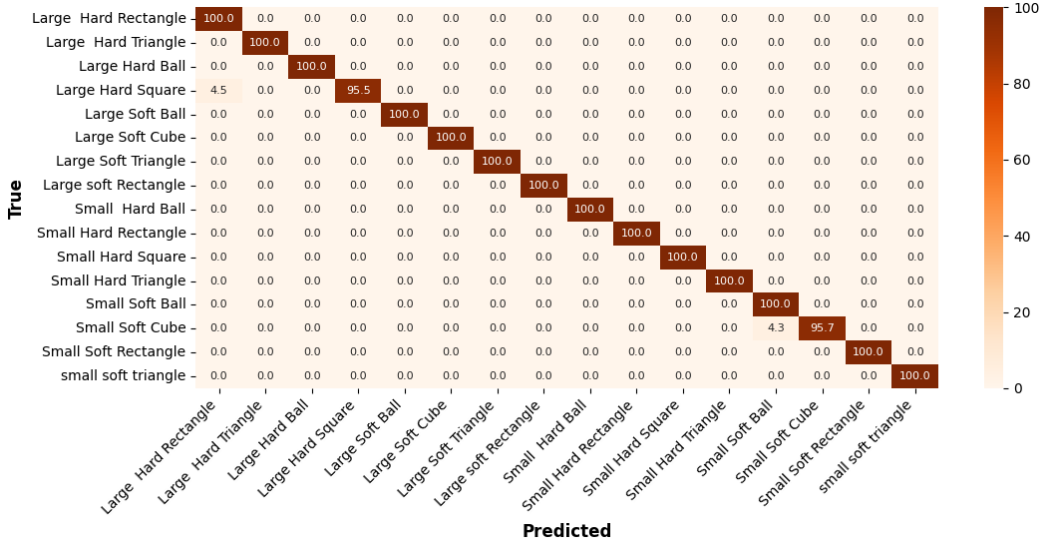
Confusion matrix of the *Printed Shapes* dataset classification performance.

The confusion matrix for the *Printed Shapes* dataset in Figure 9 shows that the model performs with very high accuracy overall. Most classes achieved 100% accuracy, indicating perfect classification. The lowest performance is observed for the *Large Hard Square* class with an accuracy of 95.5%, due to a few instances being misclassified as Large hard rectangle. This highlights a slight confusion between square and rectangle shapes, particularly when size and material differ.

### 4.2. Evaluation of Optimized Neural Network Models

This section presents a comprehensive evaluation of the optimized 1D-CNN models, focusing on both predictive performance and on-device efficiency across multiple datasets to demonstrate the robustness and generalizability of the proposed approach.

To ensure robustness and statistical relevance, the top five NAS-selected models for each dataset were evaluated. Each model underwent the proposed optimization techniques, Weight Reshaping, Quantization, and the Combined method, where the average testing accuracy (Avg Acc) across these models was calculated. The results of these evaluations are summarized in Table 3, Table 4 and Table 5, corresponding to the *Textures*, *Grasp*, and *Printed Shapes* datasets.

For the *Textures* dataset, the NAS baseline models achieved an average accuracy of 91.67%, with Weight Reshaping slightly improving this to 91.91%, Quantized NAS slightly lower at 90.96%, and the Combined method aligning with the baseline at 91.67%, reflecting stable performance across the candidate models. In the *Grasp* dataset, the NAS baseline averaged 94.44%, while Weight Reshaping reached 94.72%, Quantized NAS dropped to 90.47%. The Combined method closely matched Weight Reshaping at 94.56%, indicating consistent trends with minor variations. For the *Printed Shapes* dataset, the NAS baseline achieved 98.89% accuracy, with Weight Reshaping reaching 98.78%, Quantized NAS slightly lower at 97.72%, and the Combined method at 98.72%, showing that performance remained robust across all optimization strategies.

Overall, these results indicate that the Combined optimization technique maintains high accuracy across multiple candidate NAS models, consistently reflecting the trends observed in the top-performing model for each dataset. The patterns observed across *Textures*, *Grasp*, and *Printed Shapes* datasets confirm the robustness and generalizability of the proposed approach, demonstrating its effectiveness across different models and data conditions.

Following the evaluation of average accuracies, the top NAS-selected model for each dataset was deployed on a NUCLEO-F401RE board for on-device performance assessment. All models were converted to TensorFlow Lite format [48] and deployed via the ST Edge Developer Cloud platform [49]. Energy consumption was calculated by multiplying the power, obtained using the STM32 Nucleo Power Shield Board [50] and the STM32 Power Monitor Software (version 1.1.1) [51], by the inference time.

Before deployment, the Weight Reshape models in each dataset underwent hyperparameter tuning to recover any accuracy loss after optimization. Across all datasets, training consistently employed a batch size of 32, the Sparse Categorical Crossentropy loss function, and the Adam optimizer. The *Textures* and *Grasp* dataset models were trained with a learning rate of 0.003, whereas the *Printed Shapes* model used a learning rate of 0.001. To enhance training stability and prevent overfitting, all models incorporated early stopping based on validation loss, with a patience of 10 epochs.

The results summarized in Table 6 compare a NAS baseline model with three optimized variants across the *Textures*, *Grasp*, and *Printed Shapes* datasets. The baseline models achieved accuracies of 92.86% (*Textures*), 95.63% (*Grasp*), and 99.44% (*Printed Shapes*). Weight Reshape consistently improved these results, reaching 94.05%, 96.06% on the *Textures* and *Grasp* datasets while preserving a comparable accuracy of 99.17% on the *Printed Shapes* dataset. The Combined optimization techniques maintained the same improvements on the *Textures*, *Grasp*, and *Printed Shapes* datasets, achieving 94.05%, 96.06%, and 99.17%, respectively. In contrast, Quantized NAS showed slightly lower accuracy across all datasets, achieving 91.67% on *Textures*, 94.09% on *Grasp*, and 98.61% on *Printed Shapes*.

**Figure 10:**
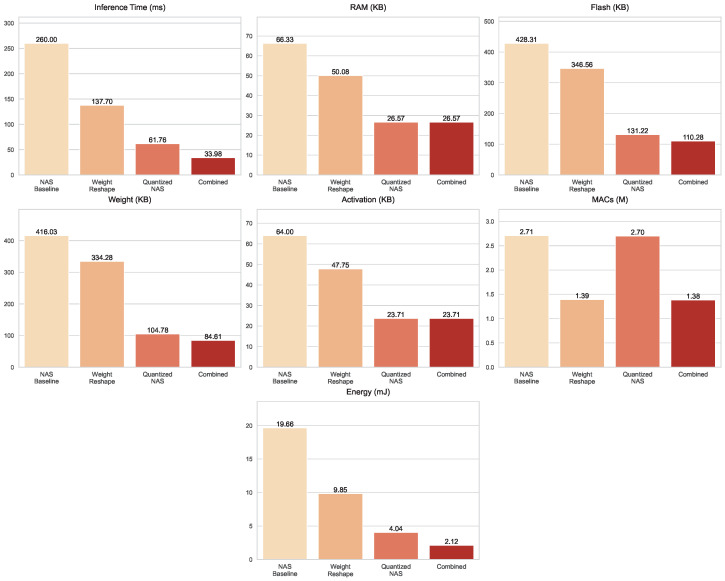
Resource usage metrics for the NAS baseline and optimized models evaluated on the *Textures* dataset.

On the *Textures* dataset, the efficiency trade-offs of each optimization strategy are summarized in Figure 10. The baseline model was the most computationally demanding, requiring 260 ms per inference, 2.709 million MACs, and 19.66 mJ of energy, with a Flash footprint of 428.31 KB. The Weight Reshape strategy substantially reduced the computational burden, halving MACs to 1.391 million, cutting latency to 137.7 ms, and lowering energy consumption to 9.848 mJ. The Quantized NAS model achieved the most aggressive storage compression, reducing Flash to 131.22 KB and weight size to 104.78 KB, while its computational demand remained relatively high at 2.698 million MACs, while energy per inference dropped to 4.037 mJ. The Combined optimization approach balanced these benefits, achieving the lowest latency at 33.98 ms, minimal storage (110.28 KB Flash, 84.61 KB weights), reduced complexity at 1.384 million MACs, and the lowest energy consumption of 2.118 mJ, making it the most practical choice for deployment.

**Figure 11:**
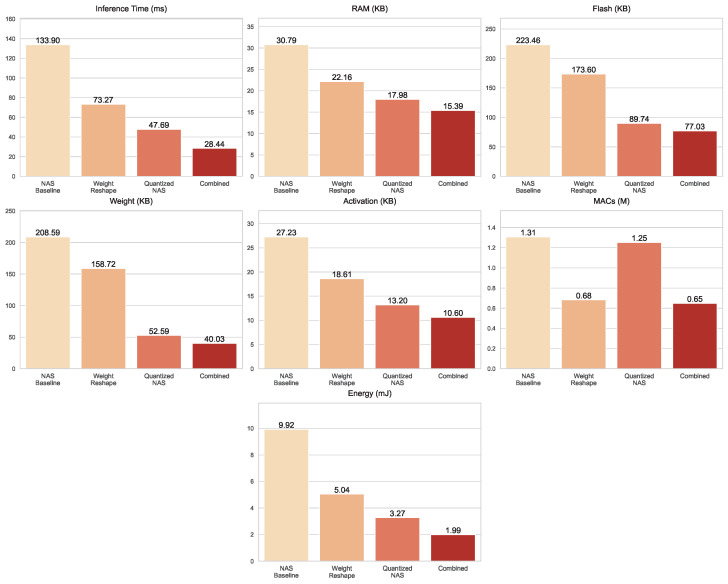
Resource usage metrics for the NAS baseline and optimized models evaluated on the *Grasp* dataset.

The *Grasp* dataset, illustrated in Figure 11, follows a similar trajectory but reveals the distinctive strengths of the optimizations. Inference time decreased progressively across the models, from 133.9 ms in the NAS baseline to 73.3 ms with Weight Reshape, 47.7 ms under Quantized NAS, and just 28.4 ms in the Combined optimization techniques. Storage demands shrank considerably as well, with Flash size reduced from 223.46 KB to 77.03 KB in the Combined variant, alongside a compact weight size of 40.03 KB. RAM usage also decreased steadily from 30.79 KB in the baseline to 15.39 KB in the Combined model. Computationally, Weight Reshape proved highly effective, cutting MACs to 0.682 million, Quantized NAS remained slightly higher at 1.251 million, while the Combined optimization techniques reached an even lower 0.647 million. Energy consumption declined in line with these improvements, dropping from 9.924 mJ in the baseline to just 1.99 mJ in the Combined model.

**Figure 12:**
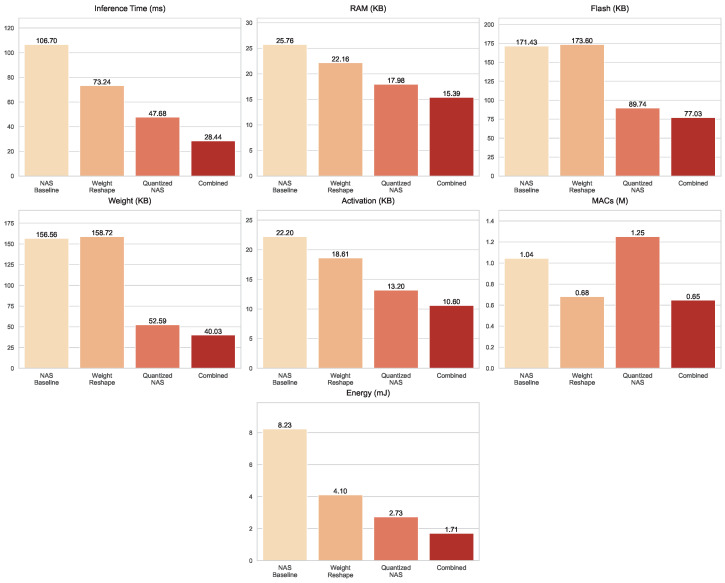
Resource usage metrics for the NAS baseline and optimized models evaluated on the *Printed Shapes* dataset.

On the *Printed Shapes* dataset, shown in Figure 12, optimization gains are most pronounced in latency and storage. The baseline inference time of 106.7 ms was reduced with Weight Reshape (73.24 ms), further dropped with Quantized NAS (47.68 ms), and was ultimately minimized in the Combined optimization techniques (28.44 ms). Memory usage followed a steady decline, with the Combined optimization techniques requiring only 15.39 KB of RAM and 10.6 KB of activations. Storage efficiency was particularly striking: while Quantized NAS already reduced Flash and weight sizes to 89.74 KB and 52.59 KB, respectively, the Combined optimization techniques compressed them further to just 77.03 KB Flash and 40.03 KB weights. For computation, Weight Reshape lowered MACs to about 0.682 million, Quantized NAS remained higher at 1.251 million, whereas the Combined optimization techniques offered the most efficient configuration at 0.647 million MACs. Energy consumption decreased progressively, from 8.231 mJ in the baseline to 4.102 mJ with Weight Reshape, 2.728 mJ with Quantized NAS, and 1.707 mJ in the Combined model.

In summary, these results demonstrate that NAS-derived 1D-CNNs can be effectively optimized for embedded deployment through strategies that balance accuracy, computational efficiency, and resource usage. Weight reshaping consistently improved or preserved both accuracy and inference performance, while quantization significantly reduced storage requirements. Although quantization can slightly increase MAC operations due to element-wise scaling, such as applying scale factors and zero points to convert between integer and floating point representations, these operations are lightweight and do not meaningfully impact inference performance. Given that power consumption is closely tied to the number and type of MAC operations, using 8-bit integer arithmetic in our quantized models drastically reduces energy compared to full-precision floating-point MACs. Consequently, even with minor increases in MACs, overall energy usage remains low. By combining weight reshaping and quantization, the models achieved the most favorable trade-offs across all datasets, reducing latency, memory usage, storage footprint, and energy consumption simultaneously. These findings illustrate a practical and cohesive approach to deploying high-performance, energy-efficient, and resource-conscious 1D-CNNs for real-world embedded AI applications.

### 4.3. Discussion

Table 7 summarizes the percentage reduction of key performance metrics achieved by the Combined optimization techniques relative to the NAS baseline across the *Textures*, *Grasp*, and *Printed Shapes* datasets. The results demonstrate that the proposed approach substantially reduces computational and memory requirements while maintaining the quality of results represented by the accuracy. Across datasets, the Combined optimization techniques show modest improvements in testing accuracy (Acc), with gains of 1.28% for the *Textures* dataset and 0.45% for the *Grasp* dataset, while the *Printed Shapes* dataset shows a slight decrease of 0.27%. This indicates that the optimization process preserves model performance and, in some cases, slightly enhances it. These findings underscore the effectiveness of integrating weight reshaping and quantization strategies with NAS, which identifies high-performing architectures and enables targeted optimization to balance efficiency and accuracy.

The computational and memory efficiency metrics show the most significant improvements. Inference time ($T_{\inf}$) is reduced by 73.3–86.9%, enabling faster predictions, while the number of MAC operations is nearly halved, reflecting a substantial decrease in computational complexity. Memory usage is also significantly reduced, with RAM requirements decreasing by up to 49.06%, and Flash memory by as much as 74.3% in the *Textures* dataset. These savings are complemented by large reductions in weight size (W), which exceed 80%, and activation size (Act), which is reduced by more than 60%. Energy consumption is also substantially lowered, with reductions of up to 89.2% in the Textures dataset. Together, these improvements highlight the efficiency of the Combined optimization techniques without compromising predictive performance. This performance analysis demonstrates that the Combined optimization techniques effectively leverage NAS in conjunction with weight reshaping and quantization to produce a network that is both highly efficient and accurate. The approach drastically reduces inference time, memory footprint, computational load, and energy consumption, while maintaining predictive accuracy.

This highlights the potential of the method for deployment on resource-constrained devices, providing a robust framework for designing neural networks that achieve an optimal balance between accuracy, computational efficiency, and energy usage. In addition, the consistency of efficiency improvements across various datasets underscores the generalization of the proposed optimization framework.

To assess the suitability of the proposed HW-NAS and the optimization pipeline for MCU deployment, the complexity of our NAS-derived 1D-CNN models and the combined optimized version was compared with representative mobile CNNs reported in the literature. The NAS baseline on the Textures dataset, for example, requires 2.7 M MAC operations, 428.31 KB Flash and 260 ms per inference while the combined optimized model reduces this to 1.384 M MACs, 110.28 KB Flash and 33.98 ms per inference. In contrast, even reduced MobileNetV2 variants with a width multiplier of 0.35 reported in literature still demand tens of millions of MACs (59.2 M) [52]. In addition, the lightweight blade damage detection model LSSD developed in [53] requires 3.541 G MACs. While a lightweight remote sensing-image-dehazing network, named LRSDN proposed in [54] requires 5.209 G MACs. Moreover, the ultra-lightweight Tiny YOLOv3 variant reported for embedded object detection demands over 1.2 G MACs [55]. These comparisons highlight the much larger computational burden of even lightweight models in the literature compared to the optimized NAS model achieved in this work, demonstrating its efficiency and suitability for MCU deployment.

Finally, the achieved results establish that the integration of NAS-derived architectures with targeted optimization strategies offers a promising pathway for deploying high-performance models in resource-constrained environments.

## 5. Conclusions

This paper introduced an optimization framework that combines HW-NAS with weight reshaping and quantization to design optimized neural classifiers for multisensory gloves. The results confirm that NAS alone can produce architectures well suited to embedded platforms, but the integration of additional optimization techniques significantly enhances efficiency without compromising accuracy. Across three diverse datasets, the combined optimization pipeline reduced latency, memory, and computational demands while preserving or improving performance, demonstrating robustness and generalization. The proposed method provides an effective way for implementing energy-autonomous wearable systems by balancing high-performance neural networks with the strict limitations of low power hardware. Beyond the presented use cases, this framework could be extended to more complex multi-modal sensing tasks, such as tactile signals with speech or other bio-signals, where efficient architectures are even more critical due to the diversity of input modalities. Moreover, coupling the optimized classifiers with energy harvesting technologies, for instance, solar energy harvesting or multi-source harvesters, would further enhance autonomy, reducing reliance on batteries and enabling long-term operation in pervasive healthcare and human–computer interaction scenarios. Together, these directions pave the way toward the development of next-generation intelligent wearables that combine accuracy and efficiency with self-sustainability and adaptability to increasingly complex multimodal environments.

## Figures and Tables

**Table 1:** Hyperparameter ranges for 1D-CNN architecture.

Hyperparameter	Ranges
No. of convolution layer	[1, 2, 3]
No. of filters per convolution layer	[8, 16, 32, 64, 128]
Kernel size	[2, 3, 4, 5]
Stride	[1]
Pooling type	[Max, Avg, None]
Pooling kernel size	[2, 3]
Activation Function	[ReLU, LeakyReLU, Tanh]
Dropout rate for convolution layer	[0.0, 0.1, 0.2]
No. of dense layers	[0, 1]
No. of neurons per dense layer	[32, 64]
Dropout rate for dense layer	[0.2, 0.3, 0.4, 0.5]
L2 regularization (λ)	[0.00001, 0.0001, 0.0005, 0.001]

**Table 2:** NAS selected 1D-CNN architectures across datasets.

Hyperparameter	Datasets		
	Textures	Grasp	Printed Shapes
No. of convolution layer	2	2	2
No. of filters per convolution layer	128	64	64
Kernel Size	2	4	4
Stride size	1	1	1
Pooling type	None	Avg	Avg
Pooling kernel size	2	2	2
Activation Function	ReLU	Tanh	Tanh
Dropout rate for convolution layer	0.0	0.2	0.2
No. of dense layers	0	0	0
No. of neurons per dense layer	64	32	32
Dropout rate for dense layer	0.4	0.4	0.4
L2 regularization (λ)	0.00001	0.001	0.001

**Table 3:** Average testing accuracy (%) for top 5 NAS selected models on the Textures dataset.

Model Rank	NAS Baseline	Weight Reshaping	Quantized NAS	Combined
Top 1	92.86	94.05	91.67	94.05
Top 2	90.48	90.48	89.29	90.48
Top 3	91.67	92.86	89.29	91.67
Top 4	92.86	91.67	91.67	91.67
Top 5	90.48	90.48	92.86	90.48
**Avg Acc**	91.67	91.91	90.96	91.67

**Table 4:** Average testing accuracy (%) for top 5 NAS selected models on the Grasp dataset.

Model Rank	NAS Baseline	Weight Reshaping	Quantized NAS	Combined
Top 1	95.63	96.06	94.09	96.06
Top 2	96.03	96.85	92.91	96.85
Top 3	92.46	94.49	92.13	93.70
Top 4	94.44	93.31	86.22	94.04
Top 5	93.65	92.91	87.01	92.13
**Avg Acc**	94.44	94.72	90.47	94.56

**Table 5:** Average testing accuracy (%) for top 5 NAS selected models on the Printed Shapes dataset.

Model Rank	NAS Baseline	Weight Reshaping	Quantized NAS	Combined
Top 1	99.44	99.17	98.61	99.17
Top 2	98.61	98.89	98.89	98.61
Top 3	97.78	97.50	95.56	97.78
Top 4	99.17	99.17	96.11	98.61
Top 5	99.44	99.17	99.44	99.44
**Avg Acc**	98.89	98.78	97.72	98.72

**Table 6:** TF-Lite testing accuracy (%) for NAS baseline and optimized models evaluated on the *Textures*, *Grasp* and *Printed Shapes* datasets.

Model	TF-Lite Testing Accuracy (%)		
	Textures Dataset	Grasp Dataset	Printed Shapes Dataset
NAS Baseline	92.86	95.63	99.44
Weight Reshape	94.05	96.06	99.17
Quantized NAS	91.67	94.09	98.61
Combined	94.05	96.06	99.17

**Table 7:** Percentage reduction (%) for the combined optimization techniques compared to NAS baseline.

Dataset	Acc	$T_{\inf}$	RAM	Flash	W	Act	MAC	Energy
Textures Dataset	1.28	86.9	59.9	74.3	79.7	63.0	48.9	89.2
Grasp Dataset	0.45	78.8	50.0	65.5	80.8	61.1	50.5	79.9
Printed Shapes Dataset	−0.27	73.3	40.3	55.1	74.4	52.3	38.1	79.3

## Data Availability

The raw data supporting the conclusions of this article will be made available by the authors on request.

## Abbreviations

The following abbreviations are used in this manuscript:

MCU	Microcontroller Unit
1D-CNN	One-Dimensional Convolutional Neural Network
HW-NAS	Hardware-Aware Neural Architecture Search
NNs	Neural Network
HQNAS	Hardware-Aware Quantized Neural Architecture Search
DJPQ	Differentiable Joint Pruning and Quantization
RL	Reinforcement Learning
EA	Evolutionary Algorithm
BO	Bayesian Optimization
CLADO	Cross Layer Dependency-Aware Optimization
MPQ	Mixed Precision Quantization
IQP	Integer Quadratic Program
FLIPS	Floating-Point and Integer Quantization Search
QAT	Quantization-Aware Training
FSR	Force-Sensing Resistor
IMU	Inertial Measurement Unit
DNNs	Deep Neural Networks
SRAM	Static Random-Access Memory
GUI	Graphical User Interface
PCB	Printed Circuit Board
I2C	Inter-Integrated Circuit
